# Complete plastome sequence of *Nepenthes mirabilis* (Nepenthaceae): a “vulnerable” herb in China

**DOI:** 10.1080/23802359.2018.1483765

**Published:** 2018-07-03

**Authors:** Zhi-Xin Zhu, Jian-Hua Wang, Chao-Rui Chen, Kun-Kun Zhao, Hua-Feng Wang

**Affiliations:** Hainan Key Laboratory for Sustainable Utilization of Tropical Bioresources, Institute of Tropical Agriculture and Forestry, Hainan University, Haikou, China

**Keywords:** *Nepenthes mirabilis*, illumina sequencing, plastome, nepenthaceae, phylogenetic analysis, caryophyllales

## Abstract

*Nepenthes mirabilis* (Nepenthaceae) is an erect or climbing (0.5–2 m tall) herb distributed in Africa (Madagascar), south and southeast Asia, North Australia and Pacific Islands (Caroline Islands). There is only one species in China. It grows in wet and sandy soils places throughout forests, grasslands, swamps, mountains, roadsides, wastelands having altitudes that sea level to 400 m. It has been ranked as a VU (Vulnerable) species in China. Here we report and characterize the complete plastid genome sequence of *N. mirabilis* in an effort to provide genomic resources useful for its conservation. The complete plastome is 155,755 bp in length and contains the typical structure and gene content of angiosperm plastomes, including two Inverted Repeat (IR) regions of 26,415 bp, a Large Single-Copy (LSC) region of 84,997 bp and a Small Single-Copy (SSC) region of 17,928 bp. The plastome contains 113 genes, consisting of 77 unique protein-coding genes, three pseudogenes, 29 unique tRNA genes, and four unique rRNA genes. The overall A/T content in the plastome of *N. mirabilis* is 62.8%. Phylogenetic analyses were performed using the entire plastome, including spacers, introns, etc., and we determined that *N. mirabilis* 32 and *Dionaea muscipula* were closely related. The complete plastome sequence of *N. 33 mirabilis* will provide a useful resource for the conservation genetics of this species as 34 well as for the phylogenetic studies in Caryophyllales.

*Nepenthes mirabilis* (Lour.) Druce (Nepenthaceae) is an erect or climbing (0.5–2 m tall) herb. It distributed in Africa (Madagascar), south and Southeast Asia, North Australia and Pacific Islands (Caroline Islands). There is only one species in China. It grows in wet and sandy soils places throughout forests, grasslands, swamps, mountains, roadsides, wastelands having altitudes from sea level to 400 m (Lu et al. 2008). It has been ranked as a VU (Vulnerable) species in China (Ministry of Environmental Protection of the People’s Republic of China and Chinese Academy of Sciences 2013). Consequently, its genetic and genomic information are urgently needed in order to promote its conservation of *N. mirabilis*. Here, we report and characterize the complete plastome of *N. mirabilis* (GenBank accession number: this study) based on Illumina paired-end sequencing data.

In this study, *N. mirabilis* was sampled from Hainan University in Hainan province of China (110.33°E, 20.06°N). A voucher specimen (Wang et al. B255) was deposited in the herbarium of the Institute of Tropical Agriculture and Forestry (HUTB), Hainan University, Haikou, China.

The modified cetyltrimethylammonium bromide (CTAB) protocol of Doyle and Doyle (1987) was used to extract genomic DNA from dry leave tissues. The genomic DNA of each sample was quantified and analyzed with Agilent 2100 BioAnalyzer (Agilent Technologies, Palo Alto, CA). Samples’ yield at least 0.8 μg DNA was selected for subsequent libraries construction and de novo sequencing. Genomic DNA of selected samples were used to build the paired-end libraries with 200–400 bp insert size. Libraries were sequenced using BGISEQ-500 platform at BGI Shenzhen, China and produced about 8 Gb high-quality per sample with 100 bp paired-end reads. Raw reads were trimmed using SOAPfilter_v2.2 with the following criteria: (1) reads with >10% base of N; (2) reads with >40% of low quality (value ≤10); (3) reads contaminated by adaptor and produced by PCR duplication. Around 6 Gb clean data for each sample were used to perform the assembling of chloroplast genome against the plastome of *Schizophragma hydrangeoides* (Genbank Accession number: KY412467) using MITObim v1.8 (Hahn et al. 2013).

Plastomes were annotated using Geneious R8.0.2 (Biomatters Ltd., Auckland, New Zealand) against the plastome of *Schizophragma hydrangeoides* (Genbank Accession number: KY412467). The annotation was corrected with DOGMA (Wyman et al. 2004). The plastome of *N. mirabilis* was found to possess a total length 155,755 bp with the typical quadripartite structure of angiosperms, containing two Inverted Repeats (IRs) of 26,415 bp separated by a Large Single-Copy (LSC) region and a Small Single-Copy (SSC) region of 84,997 and 17,928 bp, respectively. The plastome was found to contain 113 genes, including 77 protein-coding genes (six of which are duplicated in the IR), four ribosomal RNA genes, 29 tRNA genes (seven of which are duplicated in the IR) and three pseudogenes (rpoC2, *ccsA, ycf1*). Among these genes, 13 genes (*trn*A-UGC*, trn*I-GAU*, trn*K-UUU*, trn*L-UAA*, trn*V-UAC*, atp*F*, ndh*A*, ndh*B*, pet*B*, pet*D*, rpo*C1*, rpl*16*, rps*16) harboured a single intron and four genes (*rpl2, ycf3, clp*P*, rps12*) had two introns. The gene *rps12* has trans-splicing. The overall A/T content of the plastome was 62.8%, while the corresponding values of the LSC, SSC and IR regions were 64.9%, 68.9% and 57.4%, respectively.

We used RAxML (Stamatakis 2006) with 1000 bootstraps under the GTRGAMMAI substitution model to reconstruct a maximum-likelihood (ML) phylogeny of ten published complete plastomes of Caryophyllales, using *Tetragonia tetragonioides* (*Aizoaceae,* Caryophyllales) as an outgroup. The phylogenetic analysis indicated that *N. mirabilis* and *Dionaea muscipula* is closely related and all members of Caryophyllales were clustered with a high bootstrap support (BS) value (Figure 1).The *N. mirabilis* plastome reported here will provide a useful resource for the development of medicinal and edible value as well as for phylogenetic studies of Caryophyllales.

**Figure 1.**
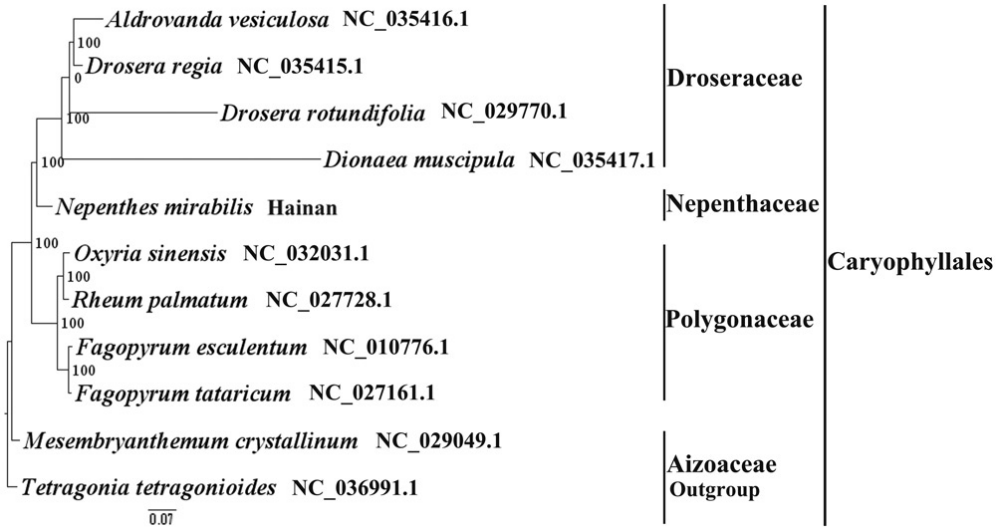
The best ML phylogeny recovered from 11 complete plastome sequences by RAxML. Accession numbers: *Nepenthes mirabilis* (this study, GenBank accession number:), *Dionaea muscipula* NC_035417.1, *Drosera rotundifolia* NC_029770.1*, Drosera regia* NC_035415.1, *Aldrovanda vesiculosa* NC_035416.1, *Oxyria sinensis* NC_032031.1, *Rheum palmatum* NC_027728.1, *Fagopyrum esculentum* NC_010776.1*, Fagopyrum tataricum* NC_027161.1, *Mesembryanthemum crystallinum* NC_029049.1, *Tetragonia tetragonioides* NC_036991.1 (lower in the figure).
